# Plant root development: is the classical theory for auxin-regulated root growth false?

**DOI:** 10.1007/s00709-021-01697-z

**Published:** 2021-09-13

**Authors:** Hans G. Edelmann

**Affiliations:** Institut für Biologiedidaktik, Universität zu Köln, Cologne, Germany

**Keywords:** Auxin (IAA), Root development, Root growth, Ethylene, Gravitropism, Auxin gradient, Lateral root, Gravitropic growth, Soil compaction, Plant signal transduction

## Abstract

One of the longest standing theories and, therein-based, regulation-model of plant root development, posits the inhibitory action of auxin (IAA, indolylacetic acid) on elongation growth of root cells. This effect, as induced by exogenously supplied IAA, served as the foundation stone for root growth regulation. For decades, auxin ruled the day and only allowed hormonal side players to be somehow involved, or in some way affected. However, this copiously reiterated, apparent cardinal role of auxin only applies in roots immersed in solutions; it vanishes as soon as IAA-supplied roots are not surrounded by liquid. When roots grow in humid air, exogenous IAA has no inhibitory effect on elongation growth of maize roots, regardless of whether it is applied basipetally from the top of the root or to the entire residual seedling immersed in IAA solution. Nevertheless, such treatment leads to pronounced root-borne ethylene emission and lateral rooting, illustrating and confirming thereby induced auxin presence and its effect on the root — yet, not on root cell elongation. Based on these findings, a new root growth regulatory model is proposed. In this model, it is not IAA, but IAA-triggered ethylene which plays the cardinal regulatory role — taking effect, or not — depending on the external circumstances. In this model, in water- or solution-incubated roots, IAA-dependent ethylene acts due to its accumulation within the root proper by inhibited/restrained diffusion into the liquid phase. In roots exposed to moist air or gas, there is no effect on cell elongation, since IAA-triggered ethylene diffuses out of the root without an impact on growth.

## Introduction

Based on the early experiments with coleoptiles of *Avena sativa* carried out by Paâl (1919) and Went (1926), the existence of “growth accelerating substances” had been postulated early last century (Went 1926; Went and Thimann 1937). The by then unidentified substance (“Wuchsstoff”), diffusible from the coleoptile tips into agar blocks, was — according to literature — soon independently identified by Went and Thimann, and named after the Greek auxein — to grow, as “auxin” (Kögl et al. 1934a, b; Went 1926; Thimann 1935). During the following decades, the relevance of auxin (IAA, indolyl-3-acetic acid) was increasingly recognised as a chemical messenger in plants (“growth substance”), relevant for a multitude of diverse physiological processes (Davies 1995; Lv et al. 2019).

Over time, other plant hormones with diverse competences have been and are still being discovered (Kleine-Vehn and Sauer 2017); in addition to what has been thought for many years, namely that plant development is regulated by a total of five hormone groups, namely auxins, gibberellins, cytokinins, abscisic acid and ethylene.

With respect to auxin, besides a broad range of multiple other research aspects, such as the apical dominance in plant shoots (Cline 1997; Kebrom 2017), or the problem of auxin transport (Gälweiler et al. 1998; Friml et al. 2002), a vast number of the investigations dealt with the principle mode of its molecular causal action responsible for cell (wall) extension in shoot parts (Cleland 1970; Edelmann et al. 1989; Kutschera 2006; Kutschera and Khanna 2020).

An early hypothesis was put forward by Hager et al. (1971, 1991), the so-called acid-growth hypothesis, leading to a longstanding debate, mainly driven by Kutschera and Schopfer (1985) and Cleland (Rayle and Cleland 1992). Despite the lack of detail still being unresolved and the still ongoing debate, the controversial discussions have nevertheless greatly contributed to the understanding of IAA-mediated cell wall bound processes in the context of cell expansion (Arsuffi and Braybrook 2018). Edelmann and Schopfer (1989) demonstrated the strict short-term interdependence between protein synthesis and cell expansion in segments of maize coleoptiles, which is inhibited within a few minutes after inhibition of protein synthesis. In contrast, even inhibition of cellulose synthesis lasting many hours has no impact on auxin-induced cell elongation (Edelmann and Schopfer 1989; Edelmann et al. 1989, 1995). With respect to the causal relevance of wall acidification in the mechanism of cell wall expansion, Cosgrove’s group eventually discovered in the 1990s the so-called expansins (McQueen-Mason et al. 1992; Cosgrove 2000). Expansins are wall proteins that are thought to mediate acid-induced growth by catalysing cell wall extension without disrupting wall polymers. Current understanding attributes expansins to a wall loosening effect without disrupting covalent bonds but rather by decreasing hydrogen bond interactions between wall polymers (Darley et al. 2001). These proteins have been thoroughly and extensively studied and have been demonstrated also to occur, at least as derivatives, in bacteria (Cosgrove 2015).

In contrast to the enhancing effect of IAA on cell expansion in upper plant organs, elongation growth of root cells is — according to the text-book — inhibited at similar concentrations (Thimann 1969). Few studies have dealt with this apparent contradiction, although effects of auxin on ethylene synthesis were demonstrated relatively early (Andreae et al. 1968; Chadwick and Burg 1970), suggesting IAA-induced inhibition of root elongation to be mediated by ethylene. Yet, other studies decisively declared IAA-induced growth inhibition of root cells not to be mediated by ethylene but by auxin itself (Eliasson et al. 1989).

Although the number of studies indicating some coherence of IAA and ethylene — and vice versa — increased (Alarcon et al. 2014), the general methodological approach of research ultimately deemed IAA the primary regulatory role of inhibition of root growth (Overvoorde et al. 2010). It is therefore not surprising, and to some extent plausible that the differential growth of graviresponding plant organs is considered as the result of a redistribution of IAA between the flanks of the organs. In graviresponding roots this results in inhibition of growth of the lower organ flank by accumulation of IAA (in contrast to graviresponding shoots, thereby accelerating growth).

The auxin (redistribution) model, generally being applied to both shoot and root growth regulation, was “not to be shaken”, although studies demonstrated that maize coleoptiles incubated in IAA solutions still reacted gravitropically. Despite a general growth-enhancing effect of the exogenous IAA, such treated organs exhibited pronounced differential growth of opposite organ flanks (Edelmann 2001). Already by then, the broadly ignored observation implied a regulation mechanism for differential cell extension independent of, or at least not based on, a gradient of IAA.

Regardless of these critical findings, the classic model has nevertheless been apparently substantiated and supported by molecular biology studies, using special markers/reporters, aimed at visualising the gravistimulated redistribution of auxin as the basic principle for differential gravitropic root growth (Ottenschläger et al. 2003a, b). For a review, also see Su et al. (2017).

From the very beginnings until nowadays, the applied experimental approach for these investigations in root growth regulation and its dependence on IAA concentration consisted for the majority of studies in incubating roots in adequate IAA solutions of different concentrations, but also by growing roots attached to vertical agar plates supplied with Murashige Skoog medium (e.g. French et al 2009). By measuring their effect on root elongation growth, as compared to water controls, the observed effects were assigned to the effect of the substance applied (e.g. Evans et al. 1980). By detecting differences in parameters or processes between the different treatments, potentially causal steps, or at least ones relevant for the mechanism of IAA-regulated cell extension growth — or inhibition of cell extension — were, and are, aimed to be elucidated (Young et al. 1990).

The logic of the present study is based on a similar principle approach: recognising differences in the dependency of the different treatments, or methodological impacts, and working out the coherent regulatory implications. Similar to the rationale of a previous study (Edelmann 2018), the validity of IAA-regulated root growth was put to the test — by comparison of differences in root behaviour in the presence of IAA as compared to water controls. Moreover, root growth was analysed during incubation in solutions of IAA as well as under conditions of exogenous IAA supply, yet with roots exposed to humid gas phase.

## Materials and methods

Maize kernels (Hybridmais, Ronaldinio, KWS) were germinated in darkness at room temperature (~ 21–23 °C) by rolling them in moistened sheets of filter paper (MN 710; 580 × 580 mm). To get straight roots, 20 kernels were placed in rows at distances of 1–1.5 cm on chromatography paper sheets (40 × 10 cm). The rolled sheets were then placed vertically in 200-mL glass beakers, and filled with distilled water to a depth of 1 cm. The beakers were then loosely covered with aluminium foil. After 2–3 days, the germinated seedlings with developed coleoptiles and roots with lengths in the range of 2 to 2.5 cm were selected for the experiments (for details, also see Hahn et al. 2006).

For measurement of root growth in liquids, seedlings were fixed between 5-mm lattice windows made of foamed material, which floated on the surface of suitable solutions in glass beakers with a volume of 300 ml, so that the roots could grow vertically downwards and shoot parts, in air, could grow upwards. The solutions were slightly aerated with air by means of a pump via silicone tubes with injection needles attached to the ends. Root growth in humid air was achieved by placing the cores of the seedlings between layers of heavily soaked soft filter paper so that the core absorbs the liquid and releases the volume of liquid to the roots and the shoot parts, i.e. the coleoptile with the primary leaf enclosed.

Graviresponding root growth was measured by two different treatments: (a) placing 2-day-old seedlings on water-imbibed filter paper on top of Styrofoam stands within PET containers (“Gerda-Dosen”) with the root horizontally free into the gas space; the container bottoms of the lidded containers were covered with water-soaked filter paper to ensure water-saturated air conditions; (b) incubation of horizontally placed roots of intact seedlings in diverse solutions in special growth chambers which allowed roots to be incubated in solutions and the rest of the seedling growing in moist air, sealing the root/shoot region with “Fitnis SH medium” (Kaniedenta), monitoring their gravitropic growth behaviour dependence on IAA concentration over time.

To measure ethylene emission from air-exposed roots as induced by exogenously supplied IAA, 3-day-old maize seedlings were incubated upside down with the grain and shoot part immersed in auxin solution in batches of 35 seedlings in glass containers and the ethylene emission determined. Ethylene was analysed with an “ETD 300” (“Sensor Sense”). Depending on head space and seedling numbers, the flow rates (stop and flow operation mode) were adjusted to an average of 1 L h^−1^, as described previously (Dreyer and Edelmann 2018).

## Results

**Fig. 1:**
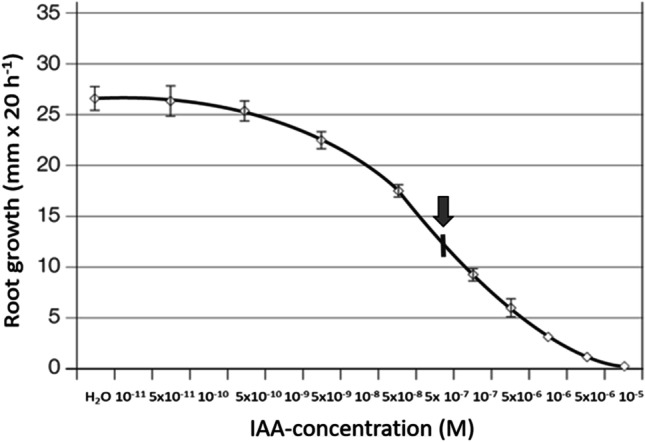
Effect of exogenous IAA (indolylacetic acid) on elongation growth of roots incubated in adequate solutions in concentrations (M/L) as indicated (values based on *n* = 30 ± SD). The vertical arrow and crossing bar indicate the IC50 value

As demonstrated in numerous previous studies (Thimann 1936; Edwards and Scott 1977; Evans and Cleland 2008), incubation of maize roots of intact seedlings in solutions of IAA results in a concentration-dependent inhibition of root elongation growth (Fig. 1). On average, above a concentration higher than 10^−10^ M IAA elongation growth is significantly inhibited, eventually ceasing at a concentration of 10^−5^ M. As shown, there is no strict linear relation between IAA concentration and inhibition of growth, but rather a sigmoidal curve, which is from a biochemical perspective, potentially indicative for some kind of cooperative mechanism. The IC50 value corresponds to a concentration of 5 × 10^−5^ M IAA.

**Fig. 2:**
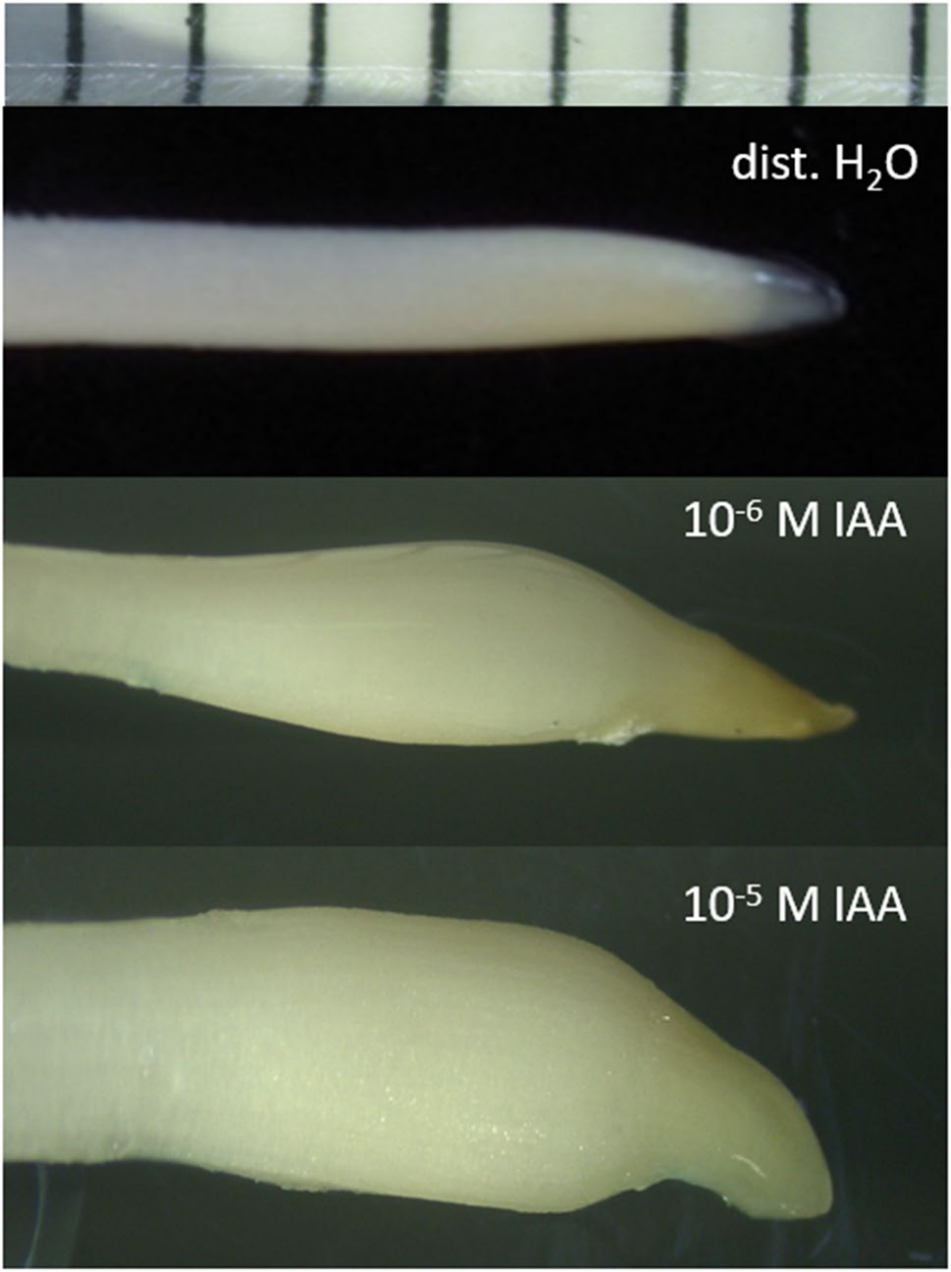
Typical images of maize root tip regions of intact seedlings incubated for 20 h in either distilled water, 10^−6^ M or 10^−5^ M IAA solution; the upper bar shows millimeteer distances

**Fig. 3:**
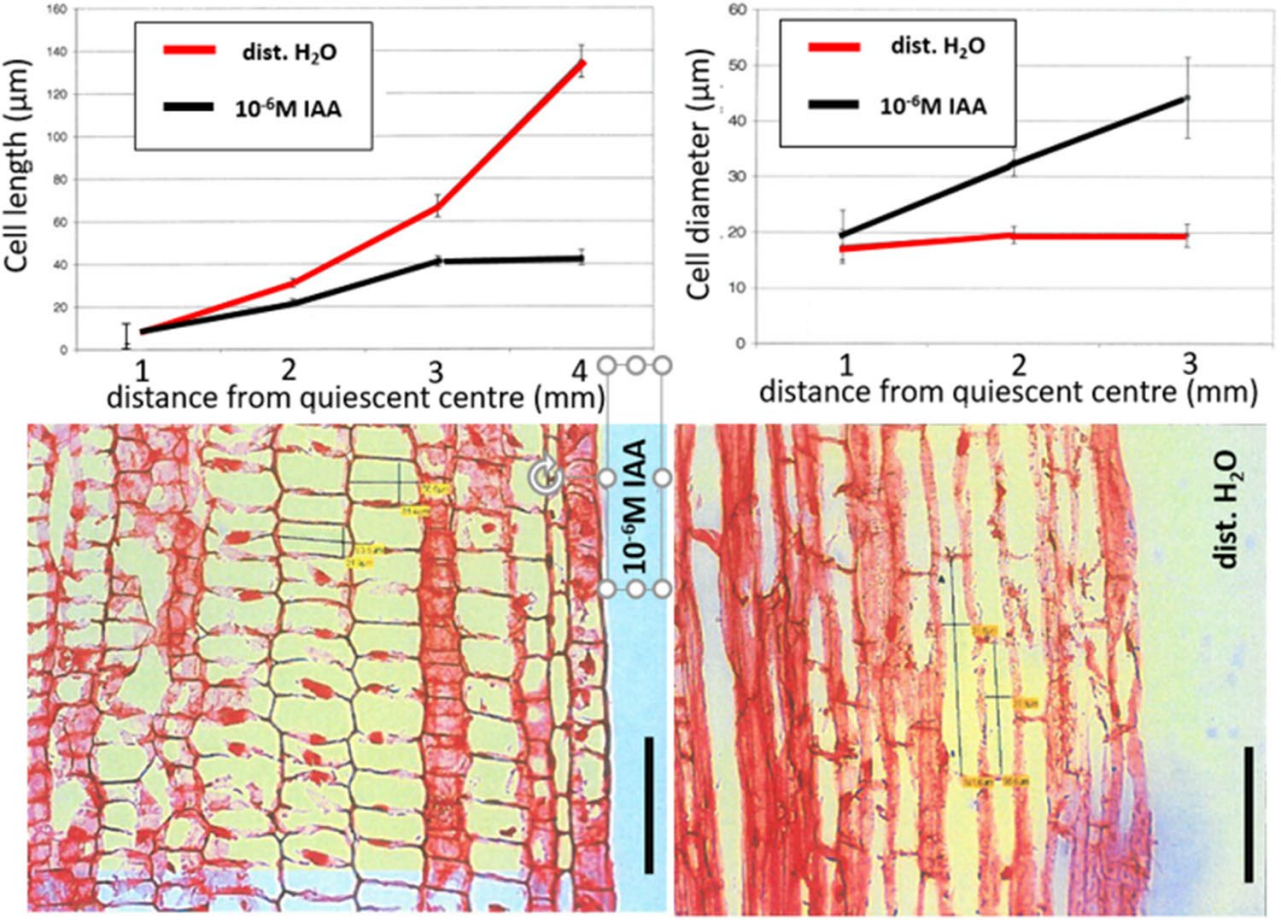
Cell length and cell diameter (upper half of the picture), as well as light microscopical images (100-fold magnification) of longitudinal sections of fixed, Eosin-stained roots of intact maize seedlings incubated for 20 h in 10^−6^ M IAA (left bottom picture) and H_2_O (right bottom picture), taken from the area of 3 mm behind the quiescent centre. Mean values (± S.E.; *n* = 18–24). Vertical bars represent 100 µm

The inhibiting effect on root elongation is at higher IAA concentrations, i.e. 10^−5^ M, but also 10^−6^ M, macroscopically characterised by root swelling (Fig. 2). This effect is accompanied by inhibited cell elongation and an increase in the cell circumference of the cells within the elongation zone of the root (Fig. 3), which is reminiscent of an effect that has been shown many times for the effect of ethylene.

**Fig. 4:**
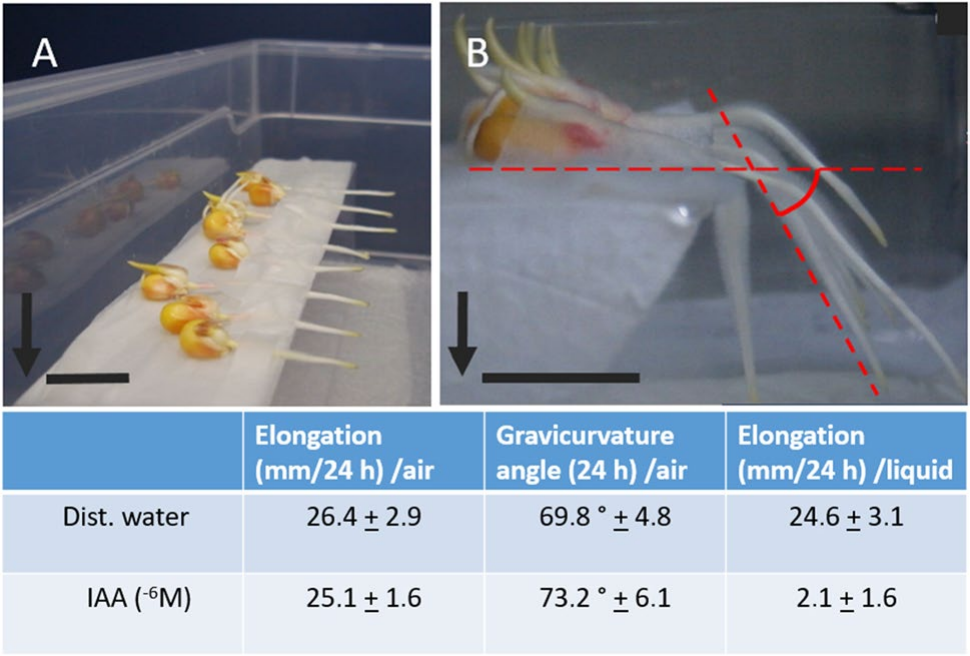
Images of the methodical procedural steps for elongation growth/gravistimulation experiments; (**A**) Three-day-old maize seedlings horizontally placed on Styrofoam bases, covered with water- or IAA solution-imbibed filter paper in water vapour-saturated plastic boxes at time = 0; (**B**) Typical graviresponses of the seedling roots after 24 h of gravistimulation. Seedlings placed on Styrofoam pedestals (with partial coverage of base parts of the roots); elongation of air exposed roots (air), gravicurvature of roots exposed to air (air), and elongation of roots incubated in solutionimbibed filter paper (liquid). Arrows indicate the gravivector; horizontal bars represent 1-cm length. Mean values (± S.E.; *n* = 24)

In contrast to these “classical observations”, this supposedly typical, root-specific IAA effect was not observed when the roots were incubated in IAA-imbibed filter paper — apart from the very first few millimetres of the tip. Such growth conditions were achieved by placing and wrapping the seedlings including parts of the upper root in solution-imbibed filter paper on top of small Styrofoam pedestals, the root-tip sections of which were allowed to grow into the water vapour-saturated air space (Fig. 4). In fact, such roots did not differ from adequate water controls, neither with respect to root elongation, nor the gravicurvature angle of the roots, obtained within a stimulation period of 20 h.

This unexpected finding animated clarification of how gravistimulated growth is affected in horizontally gravistimulated roots, incubated in solutions of IAA. In order to observe any growth (which is inhibited in solutions of 10^−5^ M IAA), we chose a concentration of 10^−6^ M IAA, which has been shown to inhibit root elongation growth by on average 87% (Fig. 1). In doing so, it turned out that roots exhibited during a period of 30-h gravitropic growth (data not shown), despite a general growth inhibition by on average 85% and several fold faint swelling of the roots (similar to the effect shown in Fig. 2). This effect is reminiscent of earlier studies in coleoptiles, in which coleoptiles of maize gravistimulated in solutions of IAA exhibited pronounced gravitropic growth, despite a — in this case — pronounced general growth stimulation (Edelmann 2001).

The inhibiting effect of IAA on root elongation growth was only observed in roots incubated in solutions — but not in air-grown roots supplied with solutions of IAA (Figs. 1 and 4). An interesting question therefore seemed to be how a (re)immersion of previously air-grown, water- and IAA solution-supplied roots affects growth. The intention was to investigate and substantiate whether the root incubation conditions determine the effect of IAA — even after an air-growing phase of the very same roots.

**Fig. 5:**
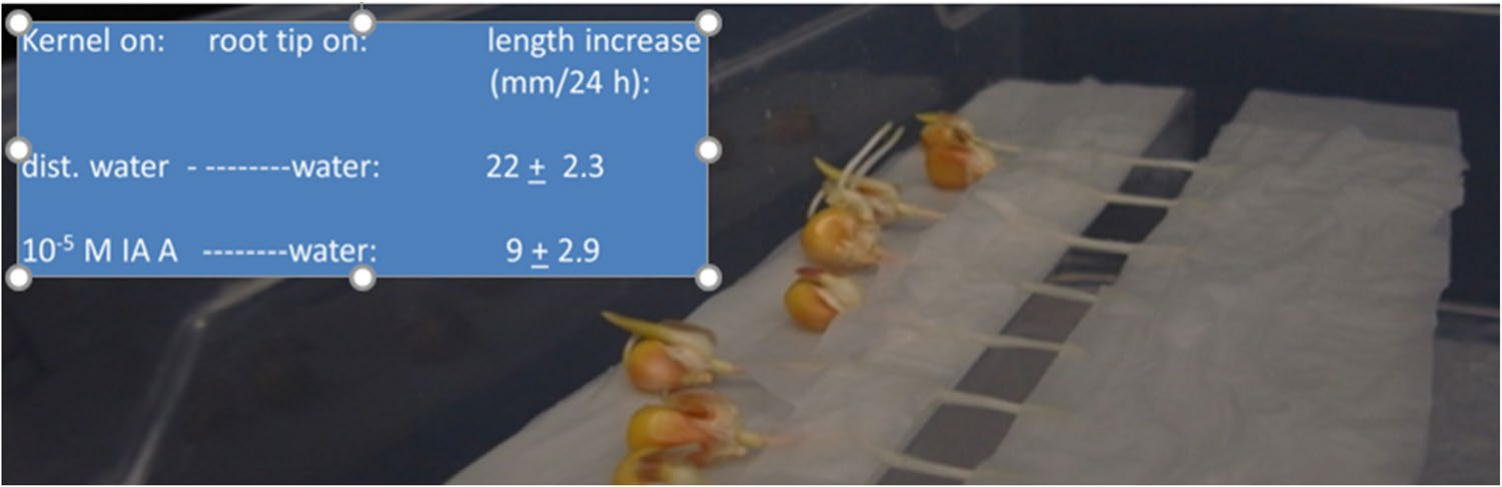
“Bridge Experiment”, in which roots of intact seedlings were supplied with liquid (water or IAA solution), grown for 8 h in moist air and then reimmersed in filter paper for following 24 h imbibed either with water or IAA solution. Mean values (± S.E.; *n* = 24)

**Fig. 6:**
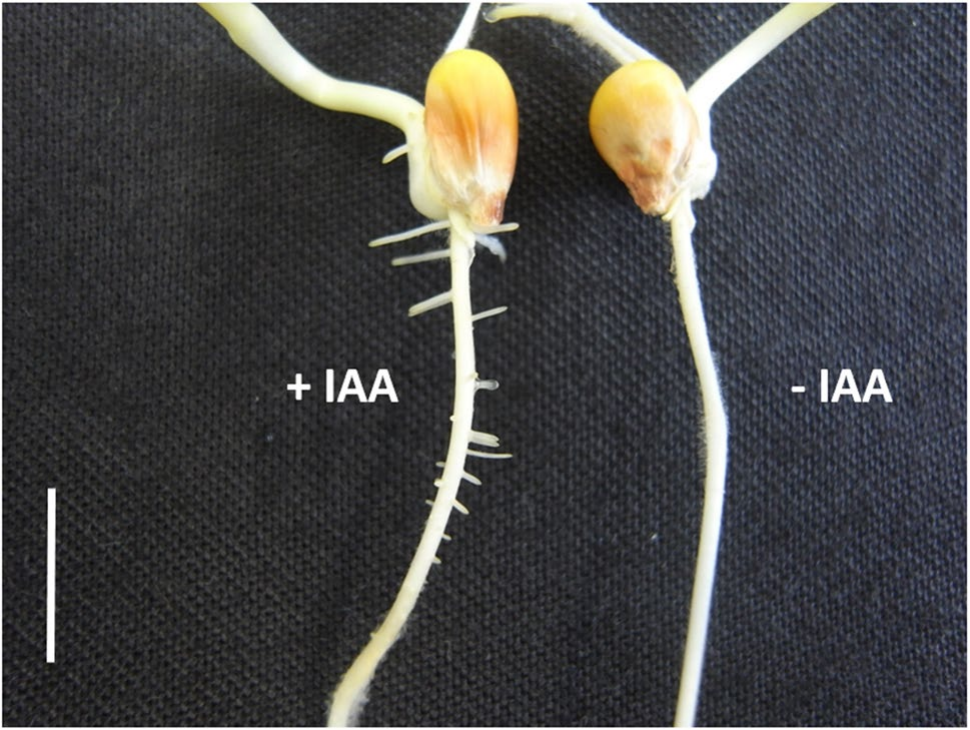
Typical images of roots of intact seedlings grown for 72 h in moist air and delivered from the shoot part with IAA solution (left hand, + IAA) or dist water (right hand, − IAA). The bar represents 1-cm length

In order to grow the very same, previously in air-grown roots in liquid, we let tips of air-grown roots of seedlings placed on small pedestals and imbibed in filter paper — both water or IAA supplied — grow into solution-imbibed filter paper (Fig. 5). It turned out that growth of air-exposed roots that were supplied with water via the shoot and the upper root area and subsequent water reimbibition was little or not impaired in comparison with air-grown roots (Fig. 5). In contrast to this, the growth of seedlings which grew with 10^−5^ M IAA over the shoot and the upper root parts and their roots in the air and then grew into water-soaked filter paper was strongly inhibited. It is therefore the incubation conditions of the roots revealing the IAA-dependant impact, which did not come to effect (Fig. 4) as long as they were not immersed in liquid phase, despite being delivered with IAA from the IAA-imbibed upper root part and residual seedling. Although this growth inhibition is an indirect proof of the presence of IAA in the root tip of seedlings which are exogenously supplied with IAA via the shoot and the upper section of the root, the absence of growth inhibition in water supplied, similarly reimmersed roots, represents strong evidence for its presence within the cells of the root tip. Although this would be diametrically opposed to any IAA transport model of recent decades (Davies 1995), it could in principle be argued that the root of the seedlings incubated in soaked filter paper, which are only supplied with IAA from the shoot and grain area, is not effectively supplied with IAA within the root proper. Evidence against such reasoning is shown in Fig. 6, where air-grown roots of water-supplied seedlings are compared to IAA-supplied roots. As already shown in many papers on the effect of ethylene (Alarcon et al. 2014), IAA causes pronounced lateral root formation (Fig. 6), which is not observed in the water control during the observed 72 h.

In a previous study in maize roots, it was shown that the more strongly ethylene synthesis was inhibited, the less pronounced the gravireaction was (Edelmann and Roth 2006). This earlier finding motivated us to test the extent to which exogenously supplied IAA induces ethylene synthesis in roots, since the IAA effects, as measured during incubation of roots in solutions reported in this study, strongly resemble ethylene effects frequently reported (Woods et al 1984; Solano and Ecker^1998^; Aloni 2021).

**Fig. 7:**
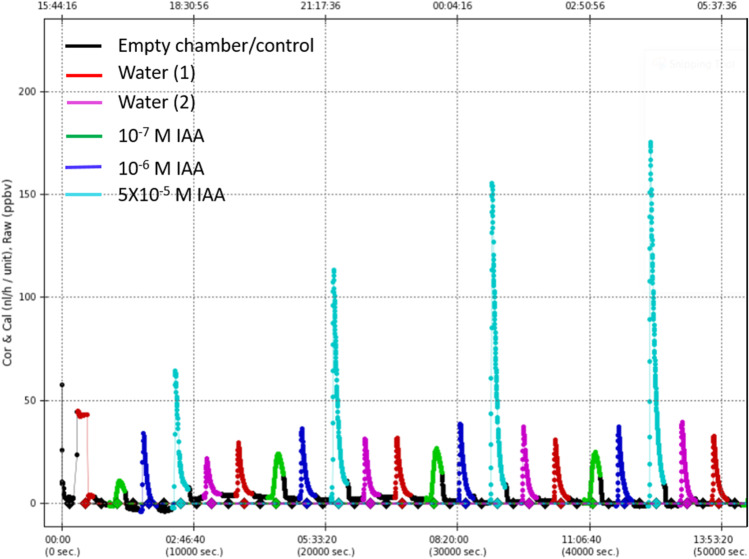
Ethylene emission from roots of 3-day-old maize seedlings incubated upside down in diverse incubation solutions/dist. water, during a measuring period of 14 h, employing the flow modulus

As shown in Fig. 7, roots of seedlings exogenously supplied with IAA via the shoot/kernel part exhibited a strong increase in emitted ethylene above a concentration of 10^−6^ M IAA over time. The original, seemingly linear correlation on dependence of the concentration of applied IAA during the first 3 h after application changed over time, with strong ethylene increases within the 5 × 10^−5^ M IAA treatment.

Also this effect demonstrates that IAA induces ethylene synthesis in roots, which was earlier shown to be crucial for its gravireaction (Edelmann and Roth 2006). Due to its solubility properties in water, under liquid incubation conditions, ethylene inevitably accumulates, which can thus exert its effect within the roots, but not so in air-exposed treatments where ethylene escapes (Fig. 7). In addition, it is known that ethylene has in water a diffusion coefficient that is 10,000 times lower than that in air (Jackson 1985; Abeles et al. 1992, Bleeker and Kende 2000). Ethylene is therefore substantially retained within the root proper during conditions of incubation in liquid. In contrast, in roots exposed in air, ethylene is not retained but to a certain extent volatilised.

## Discussion

Without any doubt auxin plays an important and crucial role in plant development, in concert with a range of various other hormones so far identified (Srivastava 2002; Kieffer et al. 2010). Concerning its role in root development, it has been reported early on since the thirties of last century to inhibit root elongation growth (Nielsen 1930; Thimann 1936). This inhibiting effect — as observed in solution-incubated roots — has since set the research framework and the research strategy for the majority of subsequent studies.

In fact, it is this key observation that has been repeatedly demonstrated in countless studies dealing with IAA-regulated root growth — it established the basis for a model on root growth regulation that has dominated research for the last hundred years (Overvoorde et al. 2010). This effect is — once more — demonstrated in Fig. 1 in this present study. It illustrates the concentration dependence of elongation growth of roots incubated in an array of solutions of IAA and distilled water.

The apparently unequivocal exogenously inducible effects of IAA, in shoots in the form of induction of cell extension growth and in roots in the form of inhibition of cell extension (Evans and Cleland 2008) soon led to two distinct main research landscapes, being characterised by different aspects of research interests (Kutschera and Schopfer 1985; Kutschera 2006; Gälweiler et al. 1998; Friml et al 2002). For long, a central problem in above ground organs consisted in the IAA-induced wall-loosening scenario, within which so-called expansins seem to play a relevant role (Cosgrove 2015). In roots, its opposing effect, namely suppression of cell extension growth, was not pursued in as much detail. The inhibiting effect on root growth by IAA mainly served to address the question of which proteins, depending on which mechanisms, accomplish IAA transport. This question has been studied particularly in dependence of gravity — not focusing on the question of the nature of inhibition of root cell growth (Young et al. 1990; Ottenschläger et al. 2003a, b). In addition to the manyfold demonstrated inhibiting effects of IAA-incubated roots, it has been shown that graviresponding roots are generally characterised by growth inhibition of the lower organ flank (Firn and Digby 1980). Therefore, most research activity during early, but also recent, years was devoted to the question of how gravitropic root growth is regulated by redistribution of the growth-inhibiting auxin — essentially ignoring other potential candidates or alternative scenarios (Friml et al. 2002). One of the crucial problems was the mechanism of the “inverse fountain model” of transport of IAA originating from the shoot part, transported within the central cylinder of the root and being reversed within the root tip to the outer epidermal tissues (Moore 2002). According to this model, in graviresponding roots, inhibition of growth of the lower organ flank is supposed to occur through a redistribution of auxin to the lower organ flank. Modern, methodically sophisticated molecular biological studies provided elegant and appealing evidence for this classic scenario (Friml et al. 2002; Ottenschläger et al. 2003a, b; Mellor et al. 2019; Brumos et al. 2018). They thereby apparently contributed to an additional consolidation, and an even more pronounced general acceptance — even if many intermediate steps in the molecular causal scenario remained open. Yet, the crucial question, namely the principle mechanism of inhibition of growth by IAA in root cells remained open. This, with respect to the aim of a coherent model, most crucial question, i.e. how does IAA inhibit cell elongation in roots, was not up for discussion: “it was a fact”. Some of the early studies on IAA inhibition of root growth however indicated ethylene induced by IAA as the causal agent (Andreae et al. 1968; Wheeler and Salisbury 1981). Also, later studies, such as by Zhao and Hasenstein (2009), substantiate the impact of IAA on ethylene. They showed that silver thiosulfate, as an ethylene inhibitor, promotes root elongation, in addition to the enhancing effect of exogenous IAA on ACC oxidases, involved in ethylene synthesis. Nevertheless, this fact and its potential relevance for the mechanism of root growth regulation have not been followed-up. One can speculate about the reasons. Employing modern, molecular biological methods, impacts or cross-talk between ethylene and IAA and vice versa were reported within the last decades (Růžička et al 2007) — yet, still attributing IAA the cardinal role for developmental root growth inhibition. This role seems to be turning out to be wrong.

The finding reported here that the widely demonstrated auxin effect on root growth no longer exists as soon as the roots are not incubated directly in solution but are supplied via the shoot tissues but also the upper root parts with highly concentrated solutions of IAA sheds new light on the physiological causal scenario and casts strong doubts on the classical model.

Counter to this conclusion, it could be argued on the one hand that IAA as supplied from the shoot as well as the upper root part (Fig. 4) is discriminated and filtered out from the liquid on the way to the elongating root tip and does not get into the volume-increasing, expanding root cells. Such a scenario would not only render previous models of auxin transport obsolete but would overturn all models originating from IAA transportation investigations. Moreover, such an argument is also refuted by a simple physiological effect illustrated in the finding that roots of seedlings exposed to moist air exhibit pronounced lateral root formation, which are, under laboratory conditions within the analysed period, not observed in water controls (Fig. 6). Such IAA effects were demonstrated in previous studies (Casimiro et al 2001; Fukaki and Tasaka 2009). This distinct effect on lateral root formation as induced by shoot-originating IAA disproves such theoretical and unphysiological considerations. It confirms similar findings in various systems by other research groups, substantiating lateral root branching as a function of IAA (Thimann 1936; Casimiro et al. 2001) as well as of ethylene (Fukaki and Tasaka 2009). Since lateral root formation as triggered by ethylene has earlier been demonstrated, the reported dependency of lateral root formation on IAA may — nevertheless — also originate from IAA-triggered ethylene, and not of IAA, as such. It would represent a function of low levels of ethylene over longer times, yet, surprisingly, without any inhibiting effect on cell elongation of the air-exposed expanding primary root. Such a causal scenario is strongly supported by the results shown in Fig. 7, which clearly demonstrates the impact of shoot-originating IAA on root ethylene emission.

On the other hand, the previously unknown dependence of IAA inhibition of root growth on external physico-chemical conditions appears in accordance with a root growth regulatory scenario, which attributes ethylene the causative role for root growth inhibition — and not auxin — despite its dependence on auxin.

To comprehend such a model, one has to consider some physiological findings and physical facts.

Although the methods were comparatively not yet very precise, early studies demonstrated IAA-induced synthesis of ethylene (Kang et al. 1971). It was therefore suggested that auxin-inhibited root growth was mediated by ethylene (Andreae et al 1968; Chadwick and Burg 1970). In fact, Kang et al. (1971) demonstrated in shoots/internodes a strong concentration-dependent correlation between IAA-mediated growth and, thereby, associated ethylene production in internode segments of *Pisum sativum*. The higher the exogenous IAA concentration, the stronger the increase in fresh weight and the extent of ethylene production. In accordance with and affirming their results, the present study demonstrates ethylene emission from roots induced by IAA applied from the kernel/shoot part of the seedling (Fig. 7). This finding clearly demonstrates the physiological impact of IAA within the root proper, apart from its effect on lateral root formation.

Looking at the circumstances and the physiological consequences of the classical methodological approach more specifically, a common feature of these studies consists in incubation of the roots in liquids. They analysed the impact of solutions of different concentrations of IAA and compared them with the ones observed in water-incubated roots — similar to the methodological approach underlying Fig. 1 in this study. By focussing on the effects of the applied hormone within the studied system, i.e. the thereby affected root metabolism, such methodical approaches apparently make sense. However, such treatments inevitably go along with effects dependent on external conditions, relevant for physiological processes within the examined system. In the presence of surrounding water, the gas diffusion conditions are quite different from those in air. As it seems, this physico-chemical effect of the surrounding water on the roots was previously either not noticed or just not considered/ignored, despite its most important physiological, as well as ecophysiological relevance.

In addition to its low solubility in water, which enhances ethylene accumulation in the root tissue, it has long been demonstrated, which ethylene has, in water, a 10-thousand fold lower diffusion coefficient as compared to air (Abeles et al. 1992). As a consequence, IAA-induced ethylene (Fig. 7) will be retained in solution-incubated roots and thereby accumulate within the root proper, in contrast to air-exposed roots which emit ethylene to diffuse away into the surrounding air space. IAA-induced ethylene and its effect on cell elongation growth of the roots, therefore, also represent a function of the aggregate state of the root environment.

The findings reported here imply IAA-induced ethylene to be retained and accumulated up to concentrations sufficient for ethylene-mediated growth inhibition. It corroborates an earlier finding by Růžička et al. (2007) who suggested — besides or in addition to IAA-induced inhibition of root growth — ethylene-mediated inhibition of root cell elongation growth, yet without realising its ineffectiveness in air-exposed roots. These authors also showed “that the effect of ethylene on root growth is largely mediated by the regulation of the auxin biosynthesis and transport-dependent local auxin distribution”; in other words, an additional one-way focus is once again attributed to the direct inhibitory action of auxin on cell elongation. Evidence for a vice versa scenario is illustrated in Fig. 6: roots exposed to high IAA concentrations in solution are characterised by root swelling, both on the organ level as well as the cell level, which is not observed in IAA-supplied roots exposed to air, which release ethylene into the air space — with no effect on root elongation.

The briefly outlined scenario in roots would provide an explanation for the seemingly contradictory effects of IAA on shoot and root cell growth — mainly because it is IAA conducted, but ethylene executed. This discrimination, which could uncritically be dismissed as trivial, has profound and lasting consequences regarding the regulation of root development processes. It involves the physical environment of the root in the cell growth regulation process, via the diffusion conditions that have a retroactive effect on the root. Ethylene obviously exerts its effect on the root not only in dependence on surrounding water conditions, but also in dependence on soil compaction conditions, as has been shown in a recent paper (Pandey et al. 2021). The plant seems not only to be able to perceive mechanical impacts, but also to be able to sense the environment, or, metaphorically speaking, get a picture of its chemical and physical environment via the degree of retention of the emitted ethylene — as a function of different external diffusion conditions. It can therefore be assumed that similarly rhizobiome-borne volatile compounds may exert a root developmental impact, apart from, or in addition to, physical soil compaction conditions (Bonkowski et al. 2000).

This finding adds to the complex networking of auxin signalling reported earlier (Paciorek and Friml 2006). It highlights an additional, most relevant feature for the execution of endogenous signalling on the dependence of the external environment, which has not yet been considered in research on root development in dependence of auxin.

Very recent evidence for inhibition of root growth dependence on external conditions, as mediated by increased ethylene due to unfavourable diffusion conditions of root-borne ethylene, was recently also presented by Pandey et al. (2021). Their findings are in strong support of the here outlined regulation scenario of IAA-triggered, ethylene-mediated inhibition of root growth in water. These authors speculate that ethylene accumulation within the root, due to soil compaction, induces “hormone responses that restrict growth” (Pandey et al. 2021). The results presented in this study, but also earlier studies (Weijers et al. 2018), strongly support inhibition of growth to be due to ethylene that inhibits growth above a certain threshold value.

As illustrated in Fig. 4, under conditions of air-exposed roots, neither elongation nor graviresponsive growth exhibits any dependence on the liquid by which roots are supplied from the shoot. Under these conditions, the only detectable difference between the two treatments, i.e. water supplied or IAA supplied, consists in the amount of emitted ethylene (Fig. 7), which obviously does not come into play because it can diffuse away. On the other hand, it has been demonstrated that gravitropic growth is gradually inhibited by increasing inhibition of ethylene synthesis (Edelmann and Roth 2006). Ethylene, and not auxin, therefore, seems to play a very subtle regulatory role in the mechanism of gravitropic growth regulation. Earlier studies (Edelmann et al. 2005) demonstrated exogenous ethylene to strongly enhance gravitropic growth of horizontally oriented maize roots, although by then its causal or regulatory positioning relative to auxin has not been recognised as illustrated in this study. Regarding the reiterated relevance of auxin in the previous graviregulation models, a crucial yet “dangerous feature” of this still fragmented model consists in its apparent plausibility: sedimentation of particles within a group of cells as an effect of gravity apparently “makes sense”, and also the redistribution of an apparent growth-inhibiting hormone, namely auxin, apparently “makes sense”. However, until now, there has no physiological process been reported causally connecting the one with the other.

The potentially intricate implications and manyfold consequences of the findings reported here for root development in general — but mainly the regulation of gravistimulated root growth — will be subject of a subsequent paper.

As recently outlined by Weijers et al. (2018), it is difficult to “describe any plant process without some direct or indirect reference to auxin”. Auxin could therefore be the indispensable director, or conductor, who also orchestrates the ethylene that then inhibits root growth — i.e. not the “direct executor”. The outcome as to whether the ethylene orchestrated by auxin comes into effect then depends on the external conditions.

With regard to a critically scrutinised, well-founded model of root growth regulation and its future exploration, the final causal addressee should not be auxin, but ethylene. That approach seems more likely to contribute to the concrete elucidation of the hitherto unsolved mechanisms of root growth regulation.
